# Diagnostic Test Accuracy of Detecting Donor‐Derived Cell‐Free DNA in Renal Transplant Rejection: A Systematic Review and Meta‐Analysis

**DOI:** 10.1155/joot/3052963

**Published:** 2026-07-28

**Authors:** Chee Siong Wong, Yegor Tryliskyy, Raja Rashid, Sowrav Barman, Vlad Shuymeko, Ajay Sharma, Mohammed Hossain

**Affiliations:** ^1^ Institute of Cancer and Genomic Sciences, University of Birmingham, Birmingham, UK, birmingham.ac.uk; ^2^ Department of Renal Transplant Surgery, Queen Elizabeth University Hospital, Birmingham, UK, nhsggc.org.uk; ^3^ Department of General Surgery, Southmead Hospital, Bristol, UK, nhs.uk; ^4^ School of Cancer and Pharmaceutical Sciences, King’s College London, London, UK, kcl.ac.uk; ^5^ Department of Renal Transplant, Queen Elizabeth University Hospital, Glasgow, UK, nhsggc.org.uk; ^6^ Department of Renal Transplant, University of Liverpool, Liverpool, UK, liv.ac.uk; ^7^ Department of Renal Transplant, Royal Free Hospital, London, UK, nhs.uk

## Abstract

**Introduction:**

Circulating donor‐derived cell‐free DNA (dd‐cfDNA) has emerged as a noninvasive biomarker for detecting allograft rejection and assessing renal status. While promising, its diagnostic test accuracy (DTA) remains unclear. This study aimed to evaluate the DTA of dd‐cfDNA in identifying rejection following renal transplantation.

**Method:**

A systematic review and meta‐analysis of DTA studies were conducted following PRISMA guidelines. Electronic databases (MEDLINE via PubMed, Embase, Scopus and Web of Science) were searched comprehensively for studies reporting the sensitivity and specificity of dd‐cfDNA in detecting renal allograft rejection. Data were analysed using Review Manager (RevMan) 5.4.1 and MetaDTA 2.0.

**Results:**

Thirteen studies were included, comprising 2214 samples across various rejection types, including antibody‐mediated rejection (ABMR) and T cell–mediated rejection (TCMR). Meta‐analysis revealed pooled sensitivities and specificities for detecting unspecified rejection of 61.1% (95% CI: 48.2%–74.8%) and 83.0% (95% CI: 76.3%–88.5%), respectively. For ABMR, pooled sensitivity increased to 75.6% (95% CI: 61.7%–86.4%) with a specificity of 82.1% (95% CI: 71.4%–88.8%). Meta‐regression analysis using a dd‐cfDNA threshold of 1% demonstrated comparable sensitivity and specificity for the detection of biopsy‐proven rejection and ABMR, yielding estimates consistent with the overall pooled results. Insufficient data were available to allow for analytical comparison in TCMR. The area under the curve (AUC) was highest in the ABMR group.

**Conclusion:**

dd‐cfDNA shows potential as a diagnostic biomarker, particularly for ABMR in renal transplantation. Further studies integrating dd‐cfDNA with biochemical and histological markers are recommended to enhance diagnostic precision.

## 1. Introduction

Cell‐free DNA (cfDNA) refers to all nonencapsulated deoxyribonucleic acid (DNA) that is free‐floating in the bloodstream. The circulating DNA is released through various biological processes, such as apoptosis and necrosis [1–3]. The application of cfDNA includes the foetal cfDNA, tumour cfDNA and donor‐derived cfDNA (dd‐cfDNA). The dd‐cfDNA accounts for a small fraction of the total cfDNA (recipient plus donor‐derived) and is detectable in the recipient’s blood and urine after solid organ transplantation (SOT) [4]. Recently, dd‐cfDNA has been studied and utilised as noninvasive plasma and urine biomarkers to monitor graft rejection in post–renal transplant recipients. Urinary cfDNA, or transrenal DNA (Tr‐DNA), is highly fragmented [5]. In comparison, the DNA molecule of tr‐DNA is shorter and less intact than that of plasma DNA [6].

When monitored longitudinally, elevated dd‐cfDNA levels are associated with a higher risk of graft rejection, ischaemia and infection [7] in SOT, including kidney, liver, heart and lung transplantation. Studies have shown that dd‐cfDNA is more sensitive and specific than serum creatinine in detecting acute rejection in patients undergoing kidney transplantation [8]. A significant breakthrough was achieved with the introduction of parallel sequencing and digital droplet polymerase chain reaction (ddPCR). dd‐cfDNA can be measured by quantitative PCR (qPCR), next‐generation sequencing (NGS) and ddPCR [9]. These technologies allowed researchers to study the effects of dd‐cfDNA on the development and maintenance of acute rejection.

Currently, standard biomarkers, such as proteinuria and serum creatinine, are the late indicators of allograft injury, often increased after significant damage has occurred [10]. Therefore, routine scheduled testing of dd‐cfDNA following kidney transplantation may provide clinicians with molecular information to help inform decision‐making. The observation of increased dd‐cfDNA in blood samples before the histologic features of graft rejection suggests a potential role for the biomarkers in reducing the number of invasive postoperative renal biopsies. However, unlike renal transplant biopsy, the role and applicability of dd‐cfDNA obtained via liquid biopsy have not been established.

### 1.1. Types of Allograft Rejection

Acute rejection of a renal transplant is classified as acute T cell–mediated rejection (TCMR) and acute antibody‐mediated rejection (ABMR). The management of these types of rejections differs because the nature of the immune system that principally mediates allograft damage is different. The most common type of acute rejection is TCMR, which occurs in 20%–25% of transplants, compared with ABMR, which occurs in around 2%–4% of transplants [11]. The hallmark of TCMR is lymphocyte infiltration, predominantly CD8 T cells, whereas the hallmark of ABMR is histologic evidence of microvascular inflammation (MVI), serologic evidence of donor‐specific antibody (DSA) and evidence of interaction with endothelium (C4d‐positive).

The Banff classification (Table 1) is an internationally recognised standard for assessing and describing the various pathological changes that can occur in a transplanted kidney. First developed during the Banff Conference in 1991 [12], the classification system grades the severity of rejection and scores the degree of inflammation, tubulitis (inflammation of tubules), intimal arteritis (inflammation of arteries) and other pathological changes in the tissue. Briefly, there are six diagnostic categories. The Banff classification is regularly updated and is available on the Banff Foundation website (https://www.banfffoundation.org/).

**TABLE 1:** Banff diagnostic categories.

Category 1: Normal biopsy or nonspecific changes
Category 2: Antibody‐mediated rejection (ABMR)
Category 3: Suspicious (borderline) for acute TCMR
Category 4: T cell–mediated rejection (TCMR)
Category 5: Interstitial fibrosis and tubular atrophy (IFTA)
Category 6: Other nonrejection changes

Source: Adapted from Roufosse C., et al., A 2018 reference guide to the Banff classification of renal allograft pathology. Transplantation, 2018. 102 (11): p. 1795–1814.

### 1.2. Clinical Application of dd‐cfDNA

Allograft unequivocally releases dd‐cfDNA into the blood, which is distinguishable from recipient‐derived cfDNA (rd‐cfDNA) [13] due to the distinctive donor genotype. This is attributed to the unique single‐nucleotide polymorphisms (SNPs) or copy‐number variations (CNVs) of each individual, which are present in the donor but absent or different in the recipient [5]. The description of quantification methods of dd‐cfDNA is beyond the scope of this review. Briefly, the NGS technique quantifies dd‐cfDNA as a proportion (%), which is calculated by using a fraction of donor‐specific and recipient‐specific sequences [14], whereas the ddPCR technique quantifies dd‐cfDNA as concentrations and reports as genomic copies of dd‐cfDNA per millilitre (mL) of patient’s plasma (cp/mL) [14, 15]. In the former technique, SNP differences and advanced bioinformatics are used to differentiate recipient and donor cfDNA. The latter determines the proportion of dd‐cfDNA when the total cfDNA concentration (dd‐cfDNA plus rd‐cfDNA) is known and also enables absolute quantification of dd‐cfDNA. In other words, both techniques can quantitatively measure dd‐cfDNA, and, more importantly, they can be performed without the need to separate recipient and donor genotypes [16]. Interestingly, a study conducted in a group of high immunological risk kidney transplant recipients has found that there was no difference in baseline dd‐cfDNA values between deceased brain death (DBD) and deceased circulatory death (DCD) [17]. Generally, any dd‐cfDNA level > 1% warrants a renal transplant biopsy, as a dd‐cfDNA level < 1% is associated with the absence of active rejection [18, 19].

### 1.3. Diagnostic Test Accuracy (DTA)

A diagnostic test is a comprehensive evaluation of a disease that includes all the tests involved in diagnosis. The gold standard is regarded as the best single test (or a combination of tests for diagnosing a specific disease). Any new diagnostic tests aiming to replace the gold standard must be validated. The extent to which a test measures what it is supposed to measure is termed the accuracy of a diagnostic test. Validity is measured by sensitivity and specificity [20]. If the new test is indeed better than the gold standard, high sensitivity and high specificity would be desirable.

### 1.4. Aims of the Meta‐Analysis

Renal transplantation is generally highly successful, especially in the short term; however, long‐term outcomes are less favourable. Although the long‐term survival of kidney allografts has significantly increased over the last few decades, the 5‐year graft survival of deceased and living donors is reported as 86.1% and 91.1%, respectively (https://ctstransplant.org/public/newsletters/2017/2017-2.html). Therapeutic drug monitoring, screening for BK viraemia and DSA, and performing surveillance biopsies are the current standard of care in monitoring transplant rejection in most transplant centres [21–23].

Conventional tests, such as serum creatinine (or estimated glomerular filtration rate), proteinuria and DSA, are noninvasive modalities for detecting allograft rejection; however, they are less sensitive. Serum creatinine is the first indicator of allograft dysfunction, but it often lags considerably, rendering it less reliable as a biomarker. Allograft biopsy is the gold standard method for detecting rejection. However, it is an invasive procedure and should be performed more selectively or, if possible, entirely avoided, in favour of a noninvasive test to detect allograft rejection. In this regard, identifying a biomarker or biomarkers that can detect graft injury or loss is key to ongoing research in this field. A more sensitive and specific assay is needed to improve care and long‐term graft function.

There has been conflicting evidence regarding the dd‐cfDNA accuracy test. Some authors advocate the measurement of this new biomarker, which seems to offer a promising noninvasive approach for detecting acute rejections [24]; however, data from other authors do not support the claim otherwise [25]. In the latter, Chang et al. found that the sensitivity was very low among surveillance biopsies and clinically indicated biopsies in detecting both rejection and ABMR [25]. The findings from the previously published meta‐analysis [9] concluded that dd‐cfDNA could play a significant role in monitoring renal transplant recipients and improving long‐term outcomes by enabling earlier and more accurate detection of rejection episodes. A larger population (or sample size) will be necessary to establish the specificity and sensitivity of the novel approach utilising dd‐cfDNA.

The review aimed to provide an overview of published studies on dd‐cfDNA and to summarise the sensitivity and specificity of novel biomarkers for detecting graft injury in renal transplantation. Our meta‐analysis builds on previous meta‐analysis [9] and contributes to a larger patient population, thereby increasing the reliability of DTA measurements. Additionally, to improve the robustness of our analysis, we included an index test analysis comparing dd‐cfDNA and creatinine assays and a meta‐regression analysis of the dd‐cfDNA cutoff.

## 2. Methodology

In accordance with the PRISMA guidelines [26], we conducted a systematic review and meta‐analysis of DTA. This review was registered with the International Prospective Register of Systematic Reviews (PROSPERO) (Registration No. CRD42024511296) and published on 13 Feb 2024 (Version 1.0). Version 1.2 was updated and published on 25 Apr 2024 (https://www.crd.york.ac.uk/PROSPERO/view/CRD42024511296).

For systematic reviews of DTA studies, there is no intervention; hence, the PIRT statement (Population, Patient or Problem; Index Test; Reference Test; and Target condition) (refer to https://www.cebm.ox.ac.uk/resources/data-extraction-tips-meta-analysis/no-intervention) was used instead of the PICO statement [27]. The conventional PICO statement [28], which stands for Patient/Population, Intervention, Comparator/Control, and Outcome(s), was modified and structured to align with our research questions and the elements used in our database searches (Table 2).

**TABLE 2:** PIRT statement.

Element	Specific example	Manuscript
Patient	Who are the patients?	Renal transplant recipients
Index test	Which test am I interested in?	Donor‐derived cell‐free DNA (dd‐cfDNA)—plasma
Reference standard	What is the reference standard that is considered the best way of diagnosing the target condition?	Serum creatinine
Target condition	What is the target condition it should diagnose?	Allograft rejection

Source: Adapted from [27].

### 2.1. Search Strategy and Study Selection

Eligible studies were sought systematically by searching all electronic databases MEDLINE via PubMed, Embase, Scopus and Web of Science. The following search terms, ‘sensitivity’, ‘specificity’, ‘renal’, ‘kidney’, ‘transplant’, ‘transplantation’, ‘cell‐free’, ‘DNA’, ‘donor‐derived’, ‘liquid biopsy’, and ‘biomarker’, were searched using Boolean operators (AND and OR). The search end date was on 13th December 2024. The eligible studies were evaluated through a web‐based platform (Covidence) that allows researchers to create and manage the production of systematic and other literature reviews. The eligible studies were included if they met the following criteria. The inclusion and exclusion criteria are listed in Table 3.

**TABLE 3:** Inclusion and exclusion criteria.

Inclusion	Exclusion
• Studies reported sensitivity and specificity outcome of dd‐cfDNA in detecting rejection post–renal transplantation	• Other solid organ transplant (i.e. nonrenal transplant, e.g. heart, lung, liver and pancreas) and stem cell transplant
• Both antibody‐mediated rejection (ABMR) and T cell–mediated rejection (TCMR) are included	• Xenotransplantation/xenograft
	• Case report/case series/case study
	• Recipient of living donor kidney transplant
	• Paediatric or children population
	• Pregnancy
	• Malignancy or carcinoma
	• Animal studies
	• Mitochondrial cfDNA (mt‐cfDNA)
	• Not published in English
	• Article identified, but manuscript not retrievable
	• Manuscript retrievable, but data not extractable

### 2.2. Study Identification and Data Extraction

The searches were not restricted by the publication year, language or publication status. Relevant abstracts and proceedings from any national conferences were sought. In addition, a manual search of the reference lists in the included studies was conducted. Two independent authors cross‐checked these articles and then discussed and resolved any differences, either through discussion or with a third reviewer. Data extraction from eligible studies is as follows: author’s last name, year of publication, sample size, type of rejection, sensitivity and specificity. Other diagnostic characteristics, such as true positives (TP), false negatives (FN), false positives (FP) and true negatives (TN), were manually calculated.

### 2.3. Quality Data Assessment

The purpose of assessing quality is to evaluate the effects of potential sources of bias on estimates of test accuracy and of hypothesised clinical sources of heterogeneity on those estimates. The assessment can guide the validity of estimates of test accuracy and the applicability of included evidence to the review question across the DTA studies. The quality of the included studies was assessed using the QUADAS‐2 tool. The QUADAS‐2 tool is a systematically developed checklist for assessing the quality of primary studies that evaluate the accuracy of diagnostic tests. In other words, the QUADAS‐2 tool is designed to analyse the applicability and risk of bias in studies focused on DTA [29]. Table 4 summarises the QUADAS‐2 risk‐of‐bias tool.

**TABLE 4:** Risk of bias and applicability judgements using the QUADAS‐2 tool (https://www.bristol.ac.uk/population-health-sciences/projects/quadas/quadas-2/).

Domain	Patient selection	Index test	Reference standard	Flow and timing
Description	Describe methods of patient selection: describe included patients (e.g. testing, presentation, intended use of index test and setting).	Describe the index test and how it was conducted and interpreted.	Describe the reference standard and how it was conducted and interpreted.	Describe any patients who did not receive the index test(s) and/or reference standard or who were excluded from the 2 × 2 table (refer to flow diagram). Describe the time interval and any interventions between index test(s) and reference standard.

Signalling questions (yes/no/unclear)	Was a consecutive or random sample of patients enrolled?	Were the index test results interpreted without knowledge of the results of the reference standard?	Is the reference standard likely to correctly classify the target condition?	Was there an appropriate interval between index test(s) and reference standard?
	Was a case–control design avoided?	If a threshold was used, was it prespecified?	Were the reference standard results interpreted without knowledge of the results of the index test?	Did all patients receive a reference standard?
	Did the study avoid inappropriate exclusions?			Did all patients receive the same reference standard?
				Were all patients included in the analysis?

Risk of bias: High/low/unclear	Could the selection of patients have introduced bias?	Could the conduct or interpretation of the index test have introduced bias?	Could the reference standard, its conduct or its interpretation have introduced bias?	Could the patient flow have introduced bias?

Concerns regarding applicability: High/low/unclear	Are there concerns that the included patients do not match the review question?	Are there concerns that the index test, its conduct, or its interpretation differ from the review question?	Are there concerns that the target condition as defined by the reference standard does not match the review question?	

QUADAS‐2 consists of four key domains. Each domain is assessed for risk of bias, and the first three include additional questionnaires addressing concerns about applicability. The key components of the QUADAS‐2 tool in assessing dd‐cfDNA for diagnosing renal transplant rejection are illustrated as follows:

• Patient Selection: Adults suspected of having renal transplant rejection.
• Index Test: dd‐cfDNA.
• Reference Standard: Allograft biopsy.
• Flow and Timing: Appropriate time interval between index test and reference standard.

Tabular and graphical displays for a completed QUADAS‐2 assessment were subsequently generated.

### 2.4. Quantitative Data Assessment and Statistical Analysis

Quantitative data assessment was analysed using Review Manager (RevMan) Version 5.4.1 and MetaDTA Version 2.0 (https://crsu.shinyapps.io/MetaDTA/) [30, 31]. A diagnostic test calculator (https://araw.mede.uic.edu/cgi-bin/testcalc.pl) was used to determine the diagnostic test characteristics, including sensitivity, specificity, TP, FN, FP and TN. Forest plots and summary receiver operating characteristic (SROC) curves were generated to measure the pooled sensitivity and specificity outcomes. Index test and meta‐regression analyses were performed. Meta‐regression is an extension of meta‐analysis that allows comparison across studies with different dd‐cfDNA cutoff values. The analysis was performed using the MetaBayesDTA online tool (https://crsu.shinyapps.io/MetaBayesDTA/). A *p* value of less than 0.05 was referred to as statistically significant throughout.

## 3. Results

### 3.1. Study Selection

**FIGURE 1:**
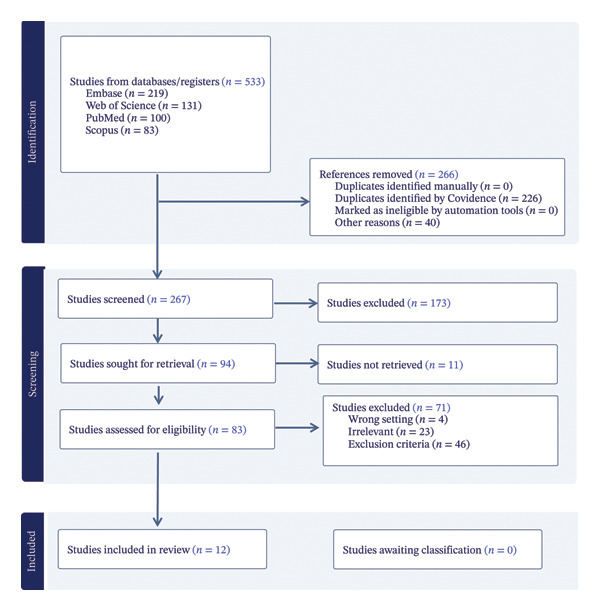
Prisma flow chart (created with 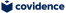).

Our review identified 533 citations, of which 13 studies were eligible for inclusion in the meta‐analysis. All were cohort studies [14, 15, 25, 32–41]. Figure 1 presents a PRISMA flow chart. We excluded abstracts presented as posters and oral presentations because no full manuscripts were available. The only exception to one abstract included [37] was that we could retrieve the data for analysis, but not for bias assessment. We have excluded studies associated with total cell‐free DNA (tcf‐DNA) [42], duplicates and other unretrievable articles. We have also excluded studies that perform dd‐cfDNA analysis of blood and urine [42–44].

### 3.2. Study Characteristics

Five were multisite dd‐cfDNA studies [32, 36, 38, 39, 41], and the rest were single‐centre cohort studies [14, 15, 25, 33–35, 40]. A total of 2214 biopsy‐matched samples were analysed with a mixture of unspecified rejection, ABMR and cell‐mediated rejection (CMR). All eligible studies were published between 2017 and 2025. The two largest patient cohorts were conducted by Bu et al. [38] and Bromberg et al. [41]. Cohort studies evaluating the performance characteristics of primary studies and diagnostic accuracy tests of dd‐cfDNA are summarised in Tables 5 and 6, respectively.

**TABLE 5:** Cohort studies evaluating performance characteristics of dd‐cfDNA to detect renal transplant rejection.

Author [ref]	Year	Study design	Number of patients (*N*)	Number of biopsies‐matched plasma	Sample type	Approach to detect dd‐cfDNA	dd‐cfDNA threshold	Performance characteristic
Bloom et al. [32]	2017	Multicentre prospective cohort study	384	107	Plasma	AlloSure®—SNPs	1.00%	Sensitivity, specificity, PPV, NPV, AUC
Jordan et al. [39]	2018	Multicentre prospective cohort study	384	61	Plasma	AlloSure®	1.00%	Sensitivity, specificity, PPV, NPV, AUC
Whitlam et al. [14]	2019	Single‐centre prospective cross‐sectional study	55	55	Plasma	Concentration	0.75% or 21cp/mL	Sensitivity, specificity, PPV, NPV, AUC
Sigdel et al. [33]	2019	Single‐centre retrospective cohort study	193	217	Plasma	SNPs	1.00%	Sensitivity, specificity, AUC
Oellerich et al. [15]	2019	Single‐centre prospective cohort study	218	417	Plasma	Concentration and fraction of the total cfDNA	0.43% or 52 cp/mL	Sensitivity, specificity, PPV, NPV, AUC
Huang et al. [34]	2019	Single‐centre prospective cohort study	352	63	Plasma	AlloSure®	1.00%	Sensitivity, specificity, PPV, NPV, AUC
Dauber et al. [35]	2020	Single‐centre prospective cohort study	29	29	Plasma	AlleleSEQR®—INDEL panel	2.70%	Sensitivity, specificity, PPV, NPV, AUC
Gielis et al. [36]	2020	Multicentre prospective cohort study	107	81	Plasma	SNPs	0.88%	Sensitivity, specificity, AUC
Kleiboeker et al. [37]	2020	N.A.	N.A.	77	Plasma	N.A.	0.69%	Sensitivity, specificity, PPV, NPV, AUC
Zhang et al. [40]	2020	Single‐centre prospective cohort study	37	37	Plasma	SNPs	1.00%	Sensitivity, specificity, PPV, NPV, AUC
Chang et al. [25]	2021	Single‐centre retrospective cross‐sectional study	261	236	Plasma	Luminex assay	1.00%	Sensitivity, specificity, PPV, NPV, AUC
Bu et al. [38]	2021	Multicentre prospective cohort study	1092	219	Plasma	AlloSure®	1.00%	Sensitivity, specificity, PPV, NPV, AUC
Bromberg et al. [41]	2025	Multicentre prospective cohort study	1743	615	Plasma	AlloSure®	1.00%	Sensitivity, specificity, PPV, NPV, AUC

Abbreviations: dd‐cfDNA, donor‐derived cell‐free DNA; NPV, negative predictive value; PPV, positive predictive value; Ref, reference; SNPs, single‐nucleotide polymorphism.

**TABLE 6:** Diagnostic accuracy test of dd‐DNA.

Author [ref]	Year	TP	FN	FP	TN	Total sample	Rejection, unspecified	No rejection	Prevalence	Sensitivity (%)	Specificity (%)	Threshold (%)	Sample type	Technique	AUC
Bloom et al. [32]	2017	16	11	12	68	107	27^∗^	80	0.252	59.0	85.0	1.00	Plasma	NGS	0.740
Sigdel et al. [33]	2019	34	4	49	130	217	38^∗^	179	0.175	88.7	72.6	1.00	Plasma	mmPCR‐NGS	0.870
Oellerich et al. [15]	2019	16	6	122	272	417	22^∗∗^	395	0.053	73.0	69.0	0.43 or ^∗^52 cp/mL	Plasma	ddPCR	0.730
Huang et al. [34]	2019	23	11	8	21	63	34^∗∗∗^	29	0.540	67.6	72.4	1.00	Plasma	NGS	0.710
Dauber et al. [35]	2020	7	1	4	17	29	8^#^	21	0.275	88.0	81.0	2.70	Plasma	qPCR	0.840
Gielis et al. [36]	2020	5	8	10	58	81	13^#^	68	0.160	38.0	85.0	0.88	Plasma	mmPCR‐NGS	0.640
Kleiboeker et al. [37]	2020	11	8	9	49	77	19^∗∗^	58	0.247	57.9	84.5	0.69	Plasma	NGS	0.850
Chang et al. [25]	2021	16	51	8	161	236	67^∗∗∗^	169	0.284	0.24	0.95	1.00	Plasma	Luminex	N.A.
Bu et al. [38]	2021	66	47	19	87	219	113	106	0.516	0.58	0.82	1.00	Plasma	NGS	0.798
Bromberg et al. [41]	2025	100	82	52	381	615	182	433	0.296	0.55	0.88	1.00	Plasma	NGS	0.789

**Author**	**Year**	**TP**	**FN**	**FP**	**TN**	**Total sample**	**ABMR**	**Not ABMR**	**Prevalence**	**Sensitivity (%)**	**Specificity (%)**	**Threshold (%)**	**Sample type**	**Technique**	**AUC**

Bloom et al. [32]	2017	13	3	15	75	107	16	91	0.150	81.0	83.0	1.00	Plasma	NGS	0.870
Jordan et al. [39]	2018	23	6	6	26	61	29	32	0.475	81.0	82.0	1.00	Plasma	NGS	0.860
Whitlam et al. [14]	2019	12	1	5	37	55	13	42	0.236	90.0	88.0	0.75 or ^∗^21 cp/mL	Plasma	ddPCR	0.920
Huang et al. [34]	2019	18	4	12	29	63	22	41	0.349	83.3	71.8	1.00	Plasma	NGS	0.820
Zhang et al. [40]	2020	16	2	5	14	37	18	19	0.486	88.9	73.7	1.00	Plasma	NGS	0.900
Chang et al. [25]	2001	8	10	15	203	236	18	218	0.076	0.46	0.93	1.00	Plasma	Luminex	N.A.
Bu et al. [38]	2021	49	26	36	108	219	75	144	0.342	0.65	0.75	1.00	Plasma	NGS	0.802

**Author**	**Year**	**TP**	**FN**	**FP**	**TN**	**Total sample**	**TCMR**	**Not TCMR**	**Prevalence**	**Sensitivity (%)**	**Specificity (%)**	**Threshold (%)**	**Sample type**	**Technique**	**AUC**

Bu et al. [38]	2021	17	21	67	114	219	38	181	0.174	0.45	0.63	1.00	Plasma	NGS	0.699

Abbreviations: ABMR, antibody‐mediated rejection; cp/mL, copies/mL; ddPCR, digital droplet polymerase chain reaction; mmPCR‐NGS, massively multiplexed PCR–next‐generation sequencing; NGS, next‐generation sequencing; Ref, reference; TCMR, T cell–mediated rejection.

^∗^Active rejection.

^∗∗^Biopsy‐proven rejection.

^∗∗∗^Any rejection.

^#^Acute rejection.

### 3.3. Methods of dd‐cfDNA Detection and Quantification

The detection of dd‐cfDNA is based on chimerism, i.e. donor cells, except identical twins, are genetically distinct from the recipient. SNPs and insertion and deletion polymorphisms (INDELs) are the two most common methods to distinguish between donor and recipient DNA. SNPs are differences of one base pair, whereas InDels are differences of more than one base pair in the genomic DNA (gDNA). Several downstream analyses of plasma cfDNA, including qPCR, ddPCR and NGS, have been utilised to detect dd‐cfDNA. With evolving advancements in molecular diagnostics, the detection of cc‐cfDNA is now possible without performing genotyping of the donor or the recipient [45].

In our meta‐analysis, most studies have used NGS to detect dd‐cfDNA. The number of SNPs varies considerably, ranging from the lowest [32], 266, to the highest [40], 56,049 targeted SNPs. Gielis et al. [36] and Sigdel et al. [33] employ 1027 SNPs and 13,392 SNPs, respectively. Only one study utilised qPCR [35], and two studies used ddPCR to quantify the dd‐cfDNA [14, 15]. Both the detection and quantification methods of ddPCR and NGS have similar analytical validity [46].

### 3.4. Quality Data Analysis (QUADAS‐2 Tool)

**FIGURE 2:**
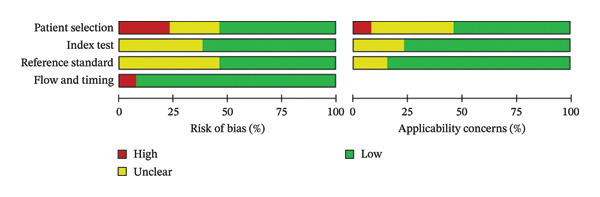
Risk of bias and applicability concerns graph: review authors’ judgements about each domain presented as percentages across included studies.

**FIGURE 3:**
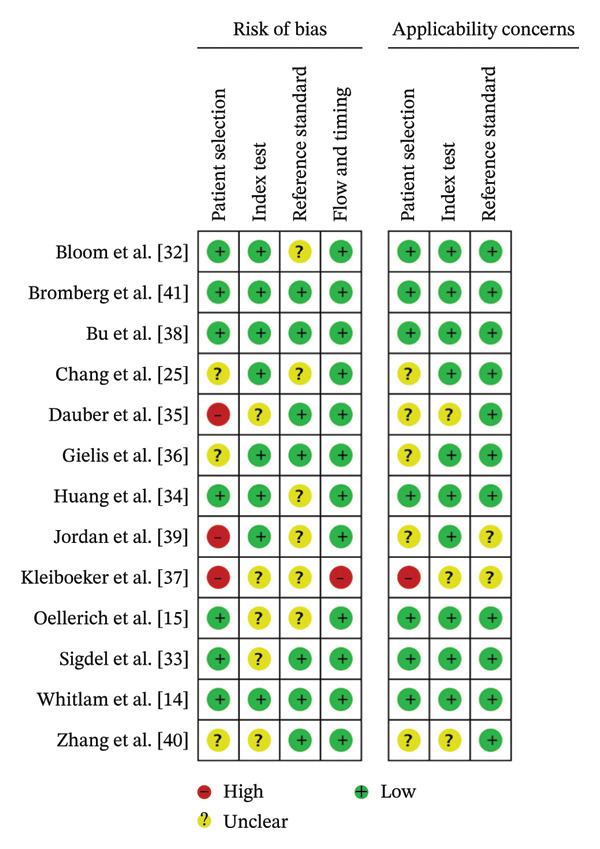
Risk of bias and applicability concerns summary: review authors’ judgements about each domain for each included study.

Overall, the majority of the studies scored low risk of bias. Two studies [35, 39] were considered to be at high risk of patient selection bias because the authors’ manuscripts did not document exclusion criteria. We could not retrieve a complete article to assess the risk of bias for one study [37]. Most of the published reports scored low risk for applicability concerns. Figures 2 and 3 present the tabular and graphical displays, respectively, of the QUADAS‐2 risk‐of‐bias assessments for all included studies.

In our study, we observed that only five studies [25, 33, 36, 38] reported that the dd‐cfDNA assay was performed at the time of biopsy, either as part of a protocol [33, 36] or as a surveillance biopsy [25, 38, 41]. The remaining studies did not clarify the temporal relationship between blood sampling and histological assessment. This weakens the correlation between dd‐cfDNA and biopsy findings. The lack of synchronisation between dd‐cfDNA testing and biopsy timing may contribute to between‐study heterogeneity, especially in sensitivity and specificity estimates (Refer to *Index Test and Meta-regression Analysis* section).

### 3.5. Quantitative Data Analysis

#### 3.5.1. Studies Reporting Diagnostic Accuracy Test of dd‐cfDNA for the Diagnosis of Graft Rejection (Unspecified Rejection)

**FIGURE 4:**
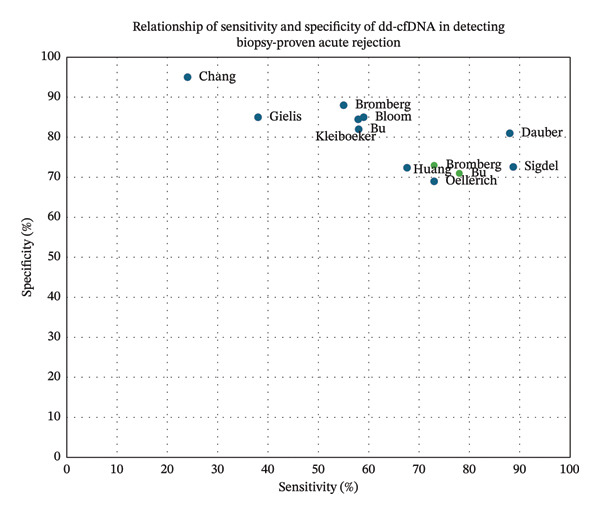
Relationship of sensitivity and specificity of dd‐cfDNA in detecting graft rejection, unspecified. The blue dots () represent the threshold of dd‐cfDNA detection at 1%, and the green dots () represent the threshold of dd‐cfDNA detection at 0.5%.

**FIGURE 5:**
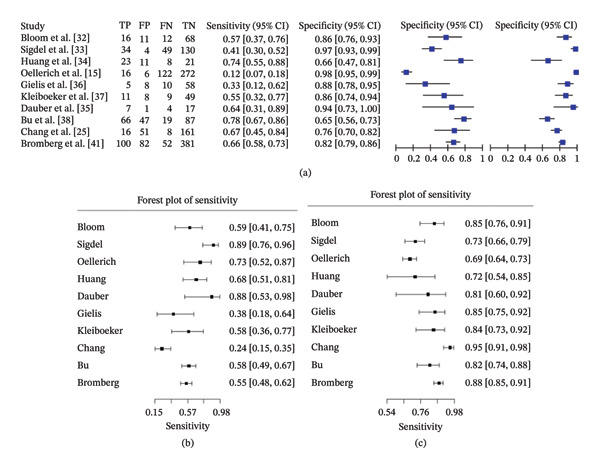
(a–c) Funnel plots depicting sensitivity and specificity of dd‐cfDNA for the diagnosis of allograft rejection (unspecified) using two methods—Review Manager (RevMan) and MetaDTA.

**FIGURE 6:**
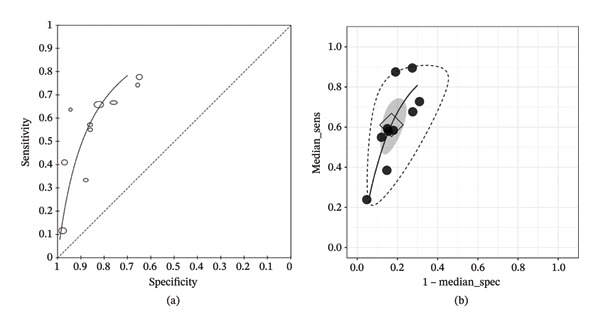
(a, b) SROC plots of dd‐cfDNA for the diagnosis of allograft rejection (unspecified) were performed using Review Manager (RevMan) and MetaDTA. The dotted line represents the 95% prediction region from the bivariate model; the greyed‐out area represents the 95% credible region from the bivariate model.

Ten studies [15, 25, 32–38, 41] were included and grouped in this domain. Four types of rejection—active rejection, biopsy‐proven rejection, any rejection and acute rejection—were identified. The relationship of sensitivity and specificity of dd‐cfDNA in detecting unspecified renal graft rejection is shown in Figure 4. The pooled sensitivity and specificity were 0.611 (95% CI, 0.482–0.748) and 0.830 (95% CI, 0.763–0.885), respectively (Figures 5(a–c)). The calculated area under the curve (AUC) was 0.77 (95% CI, 0.745–0.795), and the SROC curve is shown in Figures 6(a,b).

#### 3.5.2. Studies Reporting Diagnostic Accuracy Test of dd‐cfDNA for the Diagnosis of Graft Rejection (ABMR)

**FIGURE 7:**
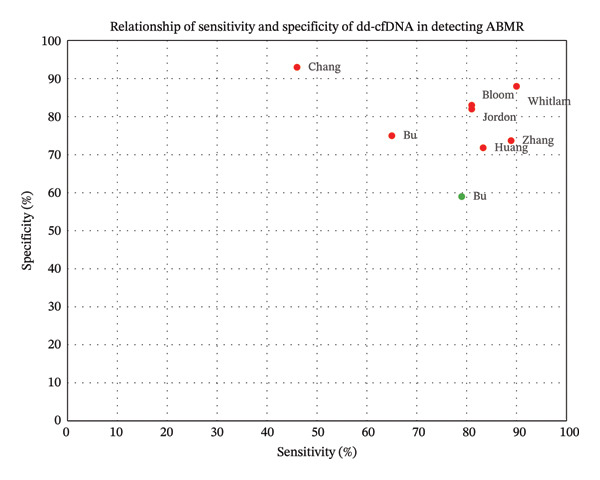
Relationship of sensitivity and specificity of dd‐cfDNA in detecting ABMR. The red dots () represent the threshold of dd‐cfDNA detection at 1%, and the green dots () represent the threshold of dd‐cfDNA detection at 0.5%.

**FIGURE 8:**
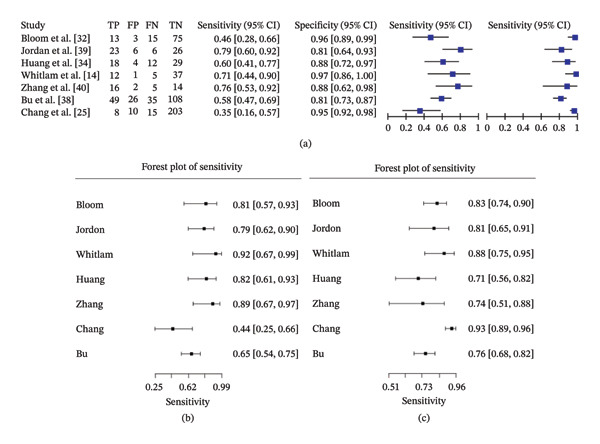
(a–c) Funnel plots depicting the sensitivity and specificity of dd‐cfDNA for the diagnosis of allograft rejection (ABMR) using two methods—Review Manager (RevMan) and MetaDTA.

**FIGURE 9:**
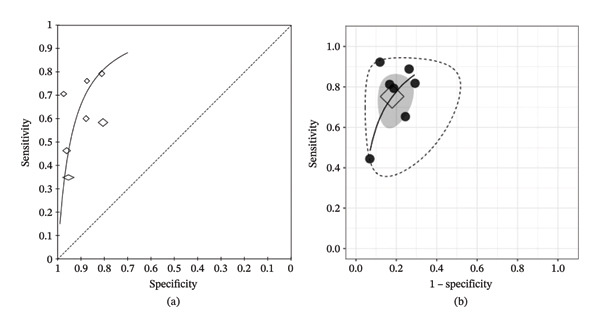
(a, b) SROC plots of dd‐cfDNA for the diagnosis of allograft rejection (ABMR) were performed using Review Manager (RevMan) and MetaDTA. The dotted line represents the 95% prediction region from the bivariate model; the greyed‐out area represents the 95% credible region from the bivariate model.

Seven studies [14, 25, 32, 34, 38–40] were included and grouped in this domain. The relationship of sensitivity and specificity of dd‐cfDNA in detecting ABMR is shown in Figure 7. The pooled sensitivity and specificity were 0.756 (95% CI, 0.617–0.864) and 0.821 (95%, CI 0.714–0.888), respectively (Figure 8(a–c)). The calculated AUC was 0.86 (95% CI, 0.825–0.895), and the SROC is shown in Figures 9(a,b).

#### 3.5.3. Studies Reporting Diagnostic Accuracy Test of dd‐cfDNA for the Diagnosis of Graft Rejection (CMR)

Only one study [38] reported specificity and sensitivity outcomes in this domain—CMR. Given only one study, a meta‐analysis was not performed. We observed that the DTA improved when the dd‐cfDNA detection threshold was set to 0.5% (Supporting Figures 1 and 2). The calculated AUC derived from the three studies [34, 38, 41] was 0.61 (0.581–0.639), and the SROC is shown in Supporting Figure 3.

#### 3.5.4. Receiver Operating Characteristic (ROC) Analysis

**FIGURE 10:**
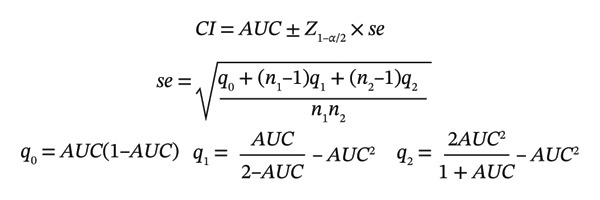
Formula for calculating 95% confidence interval for a given AUC.

The AUC, or an area under a ROC curve, measures the accuracy of a quantitative diagnostic test. We calculated the 95% CI of a given AUC using this formula (Figure 10), available on a website (https://riskcalc.org/ci/) [47].

#### 3.5.5. Index Test and Meta‐Regression Analysis

##### 3.5.5.1. Index Test Analysis of AUC

**FIGURE 11:**
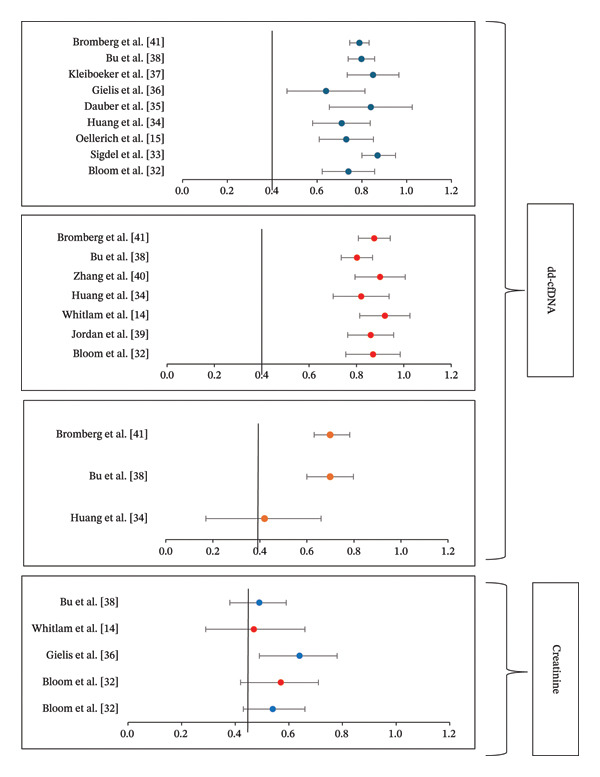
Forest plots depicting the combined AUC values of dd‐cfDNA and creatinine assays. The horizontal line represents a 95% confidence interval. Each point represents the AUC value. Blue dots () are associated with unspecified rejection, red dots () are associated with ABMR, and orange dots () are associated with TCMR.

Four studies [14, 32, 36, 38] reported the AUC for the creatinine assay, which can be combined for ROC analysis. The calculated AUC value was the highest for the dd‐cfDNA assay in detecting ABMR (0.86 [95% CI, 0.825–0.895]) and the lowest for the creatinine assay in detecting overall allograft rejection (0.54 [95% CI, 0.485–0.595]). The calculated AUCs for creatinine to discriminate rejection (unspecified) from no rejection and ABMR from no rejection were 0.56 (95% CI, 0.502–0.618) and 0.52 (95% CI, 0.403–0.637), respectively. Figure 11 summarises the ROC‐AUC results for dd‐cfDNA and creatinine assays in detecting unspecified rejection, ABMR and TCMR.

##### 3.5.5.2. Meta‐Regression Analysis of dd‐cfDNA Cutoff

Sensitivity analysis in meta‐analysis involves examining how the overall effect size estimate changes with different dd‐cfDNA threshold values. The majority of studies have utilised a 1% dd‐cfDNA threshold for rejection. In this meta‐analysis, we conducted a meta‐regression to assess the accuracy of different dd‐cfDNA cutoffs for unspecified rejection and ABMR.

**FIGURE 12:**
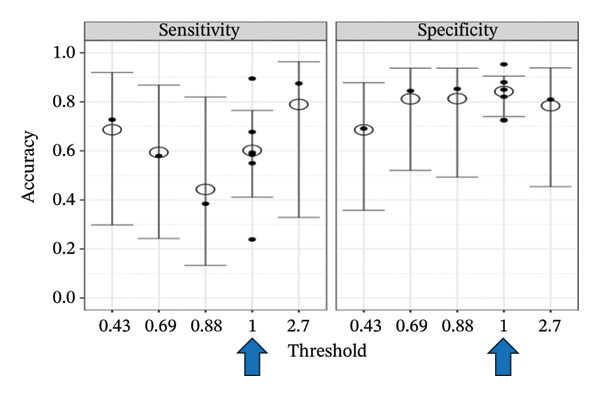
Meta‐regression analysis on different thresholds of dd‐cfDNA in detecting allograft rejection (unspecified). The vertical bars represent 95% confidence intervals of pooled studies corresponding to the dd‐cfDNA threshold. The black points represent the study‐specific data points for sensitivity (left panel) and specificity (right panel). The blue arrows represent sensitivity and specificity, which were 0.602 (95% CI, 0.411–0.765) and 0.842 (95% CI, 0.740–0.904), respectively, at a 1% dd‐cfDNA detection threshold.

**FIGURE 13:**
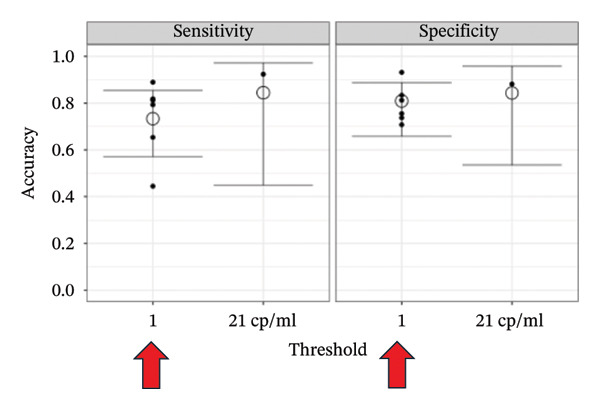
Meta‐regression analysis on different thresholds of dd‐cfDNA in detecting allograft rejection (ABMR). The vertical bars represent 95% confidence intervals of pooled studies corresponding to the dd‐cfDNA threshold. The black points represent the study‐specific data points for sensitivity (left panel) and specificity (right panel). The red arrows represent sensitivity and specificity, which were 0.729 (95% CI, 0.561–0.851) and 0.811 (95% CI, 0.688–0.886), respectively, at a 1% dd‐cfDNA detection threshold.

For a 1% dd‐cfDNA cutoff (blue arrows shown in Figure 12), the sensitivity and specificity for unspecified rejection were 0.602 (95% CI, 0.411–0.765) and 0.842 (95% CI, 0.740–0.904), respectively. With a similar cutoff of 1% (red arrows shown in Figure 13), the sensitivity and specificity for ABMR were 0.729 (95% CI, 0.561–0.851) and 0.811 (95% CI, 0.688–0.886), respectively.

**FIGURE 14:**
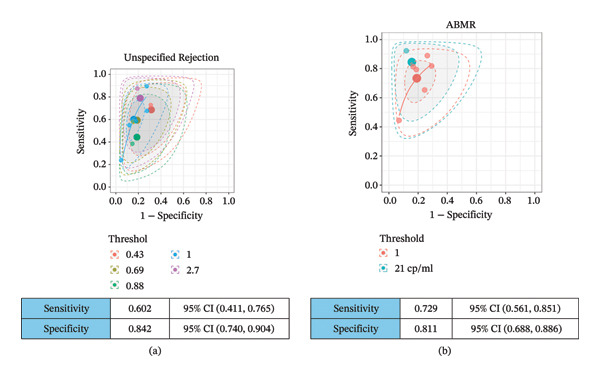
Meta‐regression—SROC plot—comparing diagnostic test accuracy of different dd‐cfDNA 1% thresholds in detecting (a) unspecified rejection and (b) AMBR.

The limited number of studies that specify concurrent dd‐cfDNA sampling and biopsy suggests a potential source of variability in diagnostic accuracy. As timing discrepancies can influence measured dd‐cfDNA levels relative to rejection events, this lack of standardisation may partly explain the heterogeneity observed across studies. A meta‐regression SROC curve with different dd‐cfDNA thresholds for unspecified rejection and ABMR is illustrated in Figure 14.

## 4. Discussion

The incidence of acute rejection in renal transplantation has been significantly decreased due to the introduction of immunosuppression medications. In general, the incidence of acute rejection is lower in living donor renal transplants than in deceased‐donor renal transplants [48]. This is likely attributed to improved compatibility in living donor transplants and a shorter cold ischaemic time (CIT) [49]. The incidence of ABMR and TCMR is based on the findings of these two systematic reviews. In one systematic review of 28 studies, the incidence of active ABMR ranged from 1.1% to 21.5%, although most studies reported an incidence of 3%–12% during the first post‐transplant year [50]. Another systematic review reported an incidence of 16% for acute TCMR in the first post‐transplant year [51].

The diagnosis of acute rejection is presently defined by histological findings through renal transplant biopsy. No specific laboratory findings can accurately diagnose rejection. Conventional tests for detecting allograft rejection are based on serial measurements of the recipient’s proteinuria and serum creatinine [10]. In transplant patients, acute rejection may be indicated by a significant increase in serum creatinine (at least 25% above baseline or a level higher than expected after transplantation) or by the presence of > 1 g/day of protein in the urine. However, serum creatinine may not immediately reflect rejection as it tends to lag behind. In addition, histological evaluation can be nonspecific and operator‐dependent. However, allograft biopsy is the gold standard for the diagnosis of AMBR and TCMR, but it is invasive and can lead to serious complications [52]. The quest for noninvasive tests that can accurately diagnose early rejection remains elusive and ongoing.

Recently, newer novel molecular tests, such as dd‐cfDNA and gene expression, have been utilised to complement conventional tests and diagnostic histological analysis in estimating the extent of allograft injury [10, 52]. Plasma dd‐cfDNA > 1% or a rising trend in serial dd‐cfDNA measurements strongly suggests graft injury due to rejection. In SOT, the presence of dd‐cfDNA in the plasma of kidney and liver transplant recipients was first demonstrated by Lo et al. in 1998 [53]. Subsequently, the utilisation of dd‐cfDNA has been clinically and analytically validated across multiple studies (see Table 7).

**TABLE 7:** Clinical studies associated with dd‐cfDNA.

DART [32]	Circulating Donor‐Derived Cell‐Free DNA in Blood for Diagnosing Active Rejection in Kidney Transplant Recipients (DART) study
ADMIRAL [38]	Assessing AlloSure® Dd‐cfDNA, Monitoring Insights of Renal Allografts with Longitudinal Surveillance (ADMIRAL) study
RADAR [54]	Resolution by AlloSure Differentiates Ambiguous Rejection (RADAR) study
KOAR [55]	Kidney Allograft Outcomes Allosure Registry (KOAR)
PROACTIVE [56]	The PROspera Kidney Transplant ACTIVE Rejection Assessment Registry
PEDAL [57]	Prospera Enhancement by Detecting dd‐cfDNA Levels (PEDAL) study

These clinical studies provide risk stratification in allograft rejection. The Circulating Donor‐Derived Cell‐Free DNA in Blood for Diagnosing Active Rejection in Kidney Transplant Recipients (DART) study was the first to analyse the donor fraction using a dd‐cfDNA method and to correlate it with rejection. In this study, Bloom et al. [32] found that at a cutoff dd‐cfDNA level > 1%, the AlloSure® assay was able to differentiate rejection from no rejection in 107 renal biopsies‐matched plasma samples (AUC = 0.74). Another study by Sigdel et al. [33] reported a higher AUC of 0.87 for the Prospera assay. Although both AlloSure® and Prospera® assays have a similar cutoff for dd‐cfDNA levels and utilise PCR‐based NGS readout, the former uses 266 SNPs and the latter uses 13,392 SNPs.

Most studies included in the meta‐analysis identify rejection at the 1% threshold. In the RADAR study, a new dd‐cfDNA threshold of 0.5% was established, and dd‐cfDNA was shown to identify subclinical allograft injury and differentiate ambiguous rejection [54]. The study demonstrates the value of dd‐cfDNA in improving kidney transplant monitoring. It also offers a more practical method for assessing the graft function. The utility of dd‐cfDNA in kidney transplant recipients is currently being evaluated in the Kidney allograft Outcomes AlloSure® Registry (KOAR, NCT03326076) [55]. In KOAR, median 100‐day dd‐cfDNA > 1% is associated with a threefold risk of adverse clinical outcomes, including rejection, detection of de novo donor‐specific antibodies (dnDSA) and return to dialysis in the first year of transplantation (HR 2.99, 95% CI 1.59–5.61; *p* < 0.005) [58]. Recently, a study called TRIFECTA brought together three complementary approaches to diagnosing kidney transplant rejection—dd‐cfDNA in blood, histopathology (Banff classification) from kidney biopsy and molecular diagnosis using the Molecular Microscope Diagnostic System (MMDx) [59]. The authors analysed the first 426 sequential indication biopsies and found that combining dd‐cfDNA fraction (%) and absolute dd‐cfDNA quantity improved the detection of active rejection compared with either metric alone.

### 4.1. dd‐cfDNA in AMBR

In patients with ABMR, dd‐cfDNA has improved diagnostic accuracy and enabled monitoring of therapeutic response. About half of DSA‐positive recipients treated with ABMR showed higher dd‐cfDNA levels than DSA‐positive AMBR‐negative recipients [60]. In other words, the presence of dd‐cfDNA correlates with AMBR in the presence of DSA. Therefore, detecting rejection early and potentially avoiding unnecessary renal transplant biopsy can be useful. On the contrary, Zhang et al. reported no significant difference between patients with DSA‐positive AMBR and those with DSA‐positive normal histology and stable graft function [40], suggesting that there is insufficient evidence to support using DSA status and dd‐cfDNA levels to predict or exclude ABMR.

### 4.2. dd‐cfDNA in TCMR

In comparison, fewer studies reported DTA in TCMR than in ABMR; therefore, the role of dd‐cfDNA levels in predicting or excluding TMCR is less robust. However, from the limited studies we have included in the meta‐analysis, the sensitivity and specificity are shown to be significantly less for TCMR than ABMR. Furthermore, in a recent systematic review and meta‐analysis, the weighted median value of dd‐cfDNA was lower in TCMR (0.27%) compared to AMBR (2.50%) [61]. Therefore, dd‐cfDNA may be useful in assessing AMBR, but it is not for TCMR.

Liquid biopsy‐based cfDNA assay clearly outperforms creatinine in detecting ABMR compared to TCMR (Figure 11), as supported by published findings from Graver et al. [13]. The molecular mechanism underlying these observations remains unknown. One possible explanation is attributed to the mechanism of allograft injury. ABMR is driven by DSAs binding to endothelial antigens, activating complement (e.g. C4d) and causing diffuse microvascular injury (glomerulitis and peritubular capillaritis). This results in widespread endothelial cell death, which releases large amounts of donor‐derived DNA into the circulation. In contrast, TCMR is primarily interstitial and tubulocentric, involving lymphocytic infiltration and focal tubular injury, which generally causes less global cell death. Unsurprisingly, the typical histologic features of renal biopsies in TCMR are characterised by inflammation of the interstitium, tubules and vessels (i, t or v) [62]. Because dd‐cfDNA reflects cell injury and necrosis, ABMR produces a stronger systemic signal.

### 4.3. dd‐cfDNA in Surveillance

The ADMIRAL study is the largest prospective cohort study that reported outcomes with dd‐cfDNA surveillance [38]. In this cohort, 1092 patients and 219 paired biopsies were followed up for three years and analysed, respectively. The study found that AlloSure® demonstrated a 62% relative improvement over serum creatinine in discriminating for all types of rejection, with the AlloSure® AUC of 0.798 and creatinine AUC of 0.492.

In the context of surveillance, Bromberg et al. [56] also demonstrated the role of dd‐cfDNA as a surveillance biomarker in kidney transplantation. Using serial measurements from the prospective ProActive Registry, the investigators showed that elevations in dd‐cfDNA preceded both ABMR and TCMR by weeks to months, whereas serum creatinine was less sensitive to early injury. These findings suggest that dd‐cfDNA may serve as a leading indicator of allograft damage, enabling earlier identification of patients at risk of rejection before overt graft dysfunction develops. Consequently, longitudinal dd‐cfDNA monitoring has the potential to complement or reduce reliance on protocol biopsies and facilitate earlier therapeutic intervention. However, further studies are required to determine whether dd‐cfDNA‐guided surveillance improves long‐term graft outcomes and is cost‐effective in routine clinical practice.

Although serial surveillance is beneficial for monitoring kidney transplants, the exact role of dd‐cfDNA testing in this setting remains unclear. We observe a longitudinal decline in plasma dd‐cfDNA levels. The utility of dd‐cfDNA in monitoring graft dysfunction in subclinical and chronic rejection has been proposed. The rationale behind this was that up to 42% of transplant recipients experienced at least one episode of subclinical rejection (subAR refers to rejection detected only during a surveillance biopsy in a setting of stable renal function) within the first 2 years following their operation [63]. This group of rejections is associated with chronic rejection and premature graft failure and loss. Therefore, noninvasive diagnostic markers for rejection are clearly needed to improve patient clinical outcomes. A study by Friedewald et al. in 2019 [64] revealed that the negative predictive value of noninvasive biomarkers was 78%–88%, making them a valuable tool for monitoring the effects of subAR treatment.

### 4.4. Time Dependency of dd‐cfDNA

The level of dd‐cfDNA changes longitudinally over time; hence, dynamic monitoring is essential. At the time of rejection, the levels of dd‐cfDNA increase gradually and decline over a period of 2 to 3 months [65]. Bunnapradist et al. [57] demonstrated that persistently high levels of dd‐cfDNA over time postrejection are associated with negative outcomes (graft loss, subsequent biopsy‐proven active rejection, postbiopsy DSA and renal dysfunction). The dynamic nature of a biomarker can be used to detect and assess the patient’s recovery from acute rejection. However, the fluctuating nature of dd‐cfDNA levels during this period suggests that using a single cutoff value across all post‐transplantation periods may be inappropriate. Although dd‐cfDNA continuously sheds into the bloodstream in allograft injury, the timing of dd‐cfDNA measurement is also crucial because the half‐life of the biomarker is only about 30 min [66]. The above variabilities would add some degree of uncertainty towards the overall accuracy of the dd‐cfDNA assay.

### 4.5. Quantification of dd‐cfDNA

When developing a diagnostic test for kidney allograft rejection using dd‐cfDNA, several factors must be considered. There are variations in dd‐cfDNA quantification and measurement methods, including fractional and absolute quantification of dd‐cfDNA levels, a relative increase in dd‐cfDNA in percentage (%) and a dd‐cfDNA threshold (%) [61]. The absolute dd‐cfDNA is measured in copies/mL. The fractional dd‐cfDNA refers to the percentage of DNA derived from donors, assuming a constant level of rd‐cfDNA. However, the level of rd‐cfDNA may vary due to various conditions, such as infection, inflammation and exercise. The variations could add heterogeneity to the data analysis. The limitation can be overcome by determining the absolute dd‐cfDNA value, thereby independently preventing variations and reducing confounding from erroneous rd‐cfDNA levels.

Using the SNP differences, the NGS method can determine dd‐cfDNA as a proportion (%) of total cfDNA [14], while ddPCR techniques can provide absolute quantification of the dd‐cfDNA or total cfDNA [14, 15]. As eluded, the detection and quantification methods of ddPCR and NGS have similar analytical validity [46]. Commercially, there are three dd‐cfDNA detection kits available: AlloSure® (CareDx), Prospera® (Natera) and Trac® (Eurofins) [67].

### 4.6. Strengths and Limitations

The first meta‐analysis, led by Xiao et al. [9], evaluates and synthesises data from various studies to determine the diagnostic accuracy of dd‐cfDNA in identifying kidney transplant rejection. The authors found that dd‐cfDNA demonstrates high diagnostic accuracy for detecting allograft rejection, offering a noninvasive alternative to traditional biopsy methods. The findings of our meta‐analysis are consistent with the previously published meta‐analysis. Additionally, our larger sample size provides more accurate results and a better population representation. To further reduce heterogeneity, we performed meta‐regression to set the dd‐cfDNA threshold at 1%. Meta‐regression restricted to studies employing a dd‐cfDNA threshold of 1% produced diagnostic performance estimates that closely mirrored the pooled analyses, suggesting that the findings were robust across studies using this commonly adopted cutoff.

The other limitations of this meta‐analysis are that we are unable to comprehensively determine the timing of biomarker acquisition, and further studies would be required to ascertain this. However, there is convincing evidence for the favourable potential of dd‐cfDNA as a biomarker for detecting ongoing graft rejection earlier—particularly for ABMR—which appears to have higher sensitivity and specificity.

One limitation of dd‐cfDNA for detecting rejection in organ transplantation is that its current application is limited to single‐organ transplants. It would be difficult to distinguish the origin of dd‐cfDNA, for example, in patients with second‐donor kidney grafts or in those with multiorgan transplants from different organ donors. No studies have reported the DTA of dd‐cfDNA from patients with a second kidney transplant or multiorgan transplant. Secondly, dd‐cfDNA cannot differentiate ABMR and TCMR. Although we would still need an allograft biopsy to confirm the type of rejection, these assays could be useful in deciding whether ongoing medical treatment is required, thereby reducing the risk of repeated biopsies.

Despite the aforementioned limitations, this assay has advantages. The assay is noninvasive and readily obtainable via routine postoperative blood tests. This would be particularly useful when a high‐risk biopsy is encountered (for example, in a patient on antiplatelet or anticoagulant therapy, uremic platelet dysfunction or when a renal transplant biopsy is difficult to obtain due to an anatomically situated renal transplant or the patient’s high BMI). Considering those clinical vignettes, these assays could mitigate the risk of a renal transplant biopsy and guide clinicians in rejection treatment, regardless of biopsy results. As with all new assays, the cost would eventually decline if the test were widely applicable and routinely used in clinical practice.

### 4.7. Future of dd‐cfDNA

Currently, renal transplant biopsy is the gold standard for diagnosing rejection [67]. dd‐cfDNA has recently emerged as a new biomarker in detecting allograft injury. However, the sensitivity and specificity of this assay are not known. A combined effect and a large population are needed to more accurately determine the sensitivity and specificity of the dd‐cfDNA assay [68]. This will allow for optimal characterisation of the test’s accuracy and improved clinical interpretation. To our knowledge, this is the largest meta‐analysis to address this question. Further studies are required to validate the utility of the dd‐cfDNA test in guiding the diagnosis and treatment of renal rejection. In particular, future diagnostic accuracy studies should standardise timing—ideally performing dd‐cfDNA sampling on the day of biopsy to ensure valid comparisons and reproducibility.

The utility of dd‐cfDNA for assessing transplant rejection has attracted widespread interest. It could help improve the decision‐making process in the clinical setting and personalise treatment. More importantly, an invasive procedure, such as a renal biopsy, could be performed more selectively or potentially avoided if the assay achieves the appropriate sensitivity and specificity to diagnose allograft rejection in renal transplantation. In addition, dd‐cfDNA is useful for excluding graft injury, thereby reducing the risk of complications associated with immediate renal transplant biopsy, such as bleeding and haematoma.

## 5. Conclusion

The meta‐analysis demonstrated that the dd‐cfDNA test has higher sensitivity and specificity for detecting AMBR than for other allograft rejections. Meta‐regression analysis estimates consistent with the overall pooled results. Furthermore, the AUC values for dd‐cfDNA are higher than those of serum creatinine in discriminating all allograft rejections. Future studies are needed to optimise the utility of dd‐cfDNA alongside other noninvasive tests in clinical practice.

## Funding

No funding was received for this manuscript.

## Disclosure

This research has been submitted, accepted and presented for oral presentation at the Transplant Society (TTS) Congress 2024 (https://journals.lww.com/transplantjournal/fulltext/2024/09001/v_111_1__diagnostic_test_accuracy_of_detecting.644.aspx) and for poster presentation at the European Society of Organ Transplantation (ESOT) Congress 2025 (https://www.frontierspartnerships.org/research-topics/184/abstract-book-22nd-congress-of-the-european-society-for-organ-transplantation).

## Conflicts of Interest

The authors declare no conflicts of interest.

## Supporting Information

Additional supporting information can be found online in the Supporting Information section.

## Supporting information


**Supporting Information** (1) Figure 1: Relationship of sensitivity and specificity of dd‐cfDNA in detecting TCMR. The orange dots () represent the threshold of dd‐cfDNA detection at 1%, and the green dots () represent the threshold of dd‐cfDNA detection at 0.5%; (2) Figure 2 (a), (b) and (c): Funnel plots depicting sensitivity and specificity of dd‐cfDNA for diagnosis of allograft rejection (TCMR) ‐ Review Manager (RevMan) and MetaDTA; and (3) Figure 3: SROC plot of dd‐cfDNA for the diagnosis of allograft rejection (TCMR) were performed using Review Manager (RevMan).

## Data Availability

The data that support the findings of this study are available in the supporting information of this article.
