# Supplementary material for: Diagnostic Test Accuracy of Detecting Donor‐Derived Cell‐Free DNA in Renal Transplant Rejection: A Systematic Review and Meta‐Analysis
Source: J Transplant. 2026 Jul 28;2026:3052963. doi: 10.1155/joot/3052963 (PMC13410283; doi:10.1155/joot/3052963)
Supplement: Supplementary file 1 — Supporting Information (1) Figure 1: Relationship of sensitivity and specificity of dd‐cfDNA in detecting TCMR. The orange dots () represent the threshold of dd‐cfDNA detection at 1%, and the green dots () represent the threshold of dd‐cfDNA detection at 0.5%; (2) Figure 2 (a), (b) and (c): Funnel plots depicting sensitivity and specificity of dd‐cfDNA for diagnosis of allograft rejection (TCMR) ‐ Review Manager (RevMan) and MetaDTA; and (3) Figure 3: SROC plot of dd‐cfDNA for the diagnosis of allograft rejection (TCMR) were performed using Review Manager (RevMan). [file JOOT-2026-3052963-s001.docx]

**SUPPLEMENTARY – FIGURES**

**Figure 1:** Relationship of sensitivity and specificity of dd-cfDNA in detecting TCMR. The orange dots (•) represent the threshold of dd-cfDNA detection at 1%, and the green dots (•) represent the threshold of dd-cfDNA detection at 0.5%.

a

**
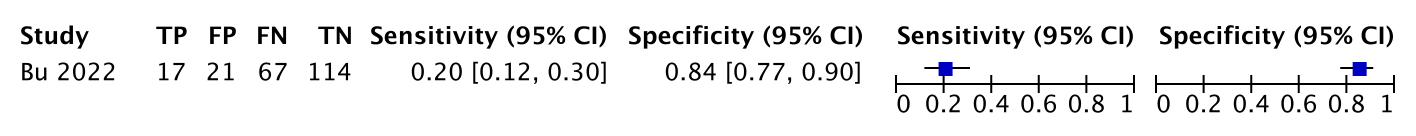
**

c

b

**Figure 2 (a), (b) and (c):** Funnel plots depicting sensitivity and specificity of dd-cfDNA for diagnosis of allograft rejection (TCMR) - Review Manager (RevMan) and MetaDTA.

**
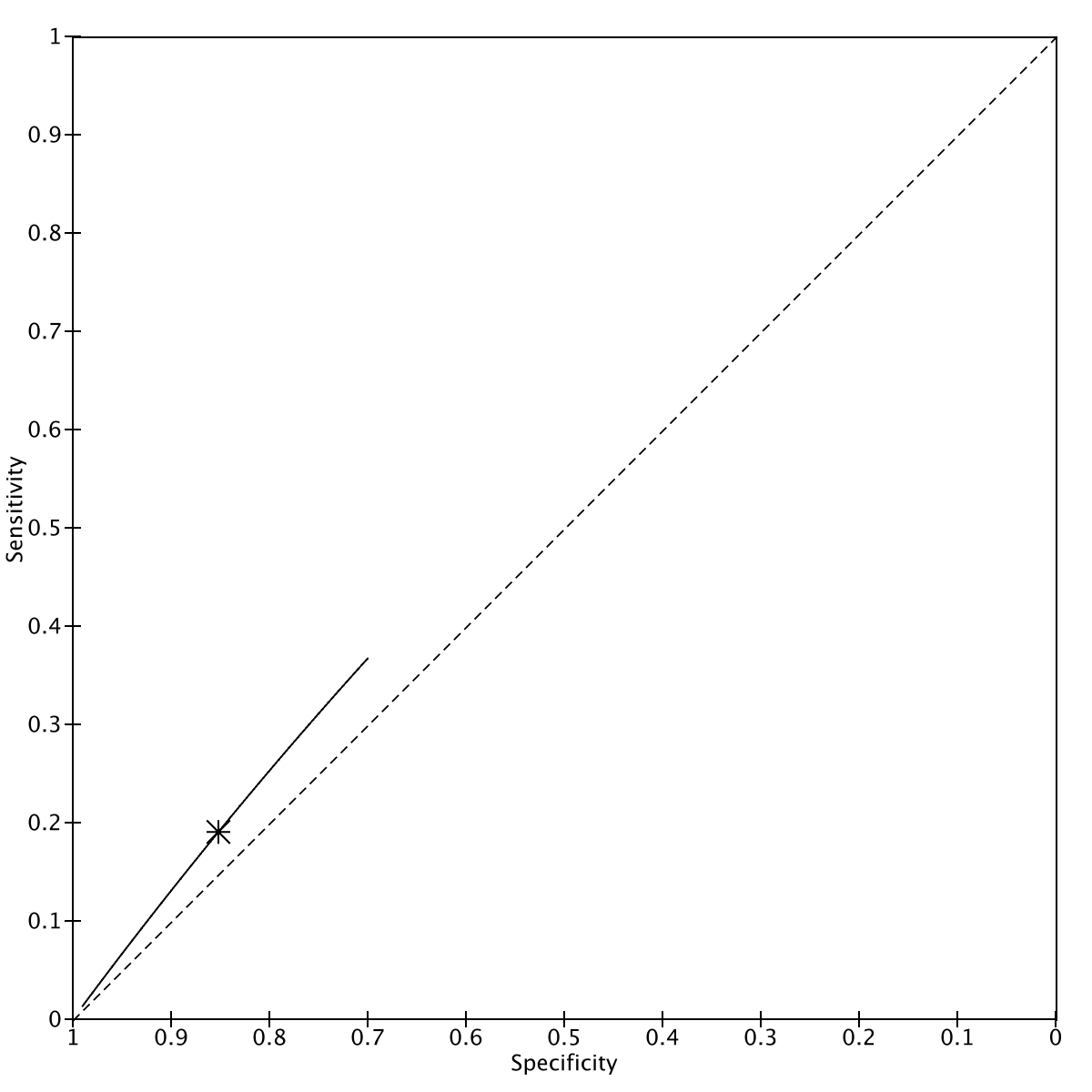
**

**Figure 3:** SROC plot of dd-cfDNA for the diagnosis of allograft rejection (TCMR) were performed using Review Manager (RevMan).
